# Facilitators and barriers to participation in health mothers’ groups in improving maternal and child health and nutrition in Nepal : A mixed-methods study

**DOI:** 10.1186/s12889-022-13859-6

**Published:** 2022-09-01

**Authors:** Ajay Acharya, Chia-Lun Chang, Mario Chen, Amy Weissman

**Affiliations:** 1 Family Health International (FHI 360), Anamika Galli Ward-4 Baluwatar, Kathmandu, Nepal; 2 Independent Researcher, Taichung, Taiwan; 3 FHI 360, Global Health, Population and Nutrition, NC Durham, US; 4 FHI 360, Asia Pacific Regional office, Bangkok, Thailand

**Keywords:** Female community health volunteers (FCHVs), Health Mother’s Group, Health and nutrition, Nepal, Women’s groups

## Abstract

**Background:**

In Nepal, Health Mother’s Groups (HMG) are women’s group-based programmes for improving maternal and child health. However, they remain underutilised with only 27% of reproductive-aged women participating in an HMG meeting in 2016. This study aimed to understand the facilitators and barriers to HMG meeting participation.

**Methods:**

We conducted a convergent mixed-methods study using cross-sectional quantitative data from the 2016 Nepal Demographic and Health Survey and primary data collected via 35 in-depth interviews and eight focus group discussions with 1000-day women and their family members, female community health volunteers (FCHVs) and health facility staff in two geographies of Nepal, Kaligandaki and Chapakot. Quantitative data were analysed using logistic regression and qualitative data using deductive coding. The results were triangulated and thematically organised according to the socio-ecological model (SEM).

**Results:**

Facilitators and barriers emerged across individual, interpersonal and community levels of the SEM. In the survey, women with more children under five years of age, living in a male-headed household, or in rural areas had increased odds of HMG participation (p < 0.05) while belonging to the Janajati caste was associated with lower odds of participation (p < 0.05). Qualitative data helped to explain the findings. For instance, the quantitative analysis found women’s education level associated with HMG participation (p < 0.05) while the qualitative analysis showed different ways women’s education level could facilitate or hinder participation. Qualitative interviews further revealed that participation was facilitated by women’s interest in acquiring new knowledge, having advanced awareness of the meeting schedule and venue, and engagement with health workers or non-government organisation staff. Participation was hindered by the lack of meeting structure and work obligations during the agricultural season.

**Conclusions:**

To improve women’s participation in HMGs in Nepal, it is necessary to address factors at the SEM’s individual, interpersonal, and community levels, such as enhancing FCHV literacy, providing advance notice of the meeting schedule, upgrading the meeting venues and reducing women’s workload through family support, particularly during agricultural season. These improvements are essential for strengthening effective implementation of HMG meetings and similar women’s group-based platforms, and for ultimately improving maternal and child health in Nepal.

## Introduction

In low and middle-income countries (LMICs), women’s groups are a recognised strategy for improving maternal and child health and are commonly used by government and development partners to deliver health and nutrition services [1, 2]. A review of seven randomised trials showed that women’s groups reduced maternal and neonatal mortality in low resource settings [2]. Similarly, another review of 36 studies in South Asia found that women groups have the potential to address multiple poor nutrition determinants through a single platform [1].

In Nepal, health mother’s groups (HMGs) are women’s groups that have operated since 2010 to address poor maternal and child health outcomes. In 2016, more than half of under-five children (53%) were anaemic, 36% were stunted, and 27% were underweight, while 41% of reproductive age women were anaemic and nearly 1 in 5 (17%) were underweight [3]. HMGs are important health and nutrition services delivery platforms in communities that may improve these indicators. HMGs target all interested reproductive-age women, though women in the 1000 days (from conception to the child’s second birthday) and mothers from marginalised communities are particularly encouraged to participate. HMGs have a minimum of 11–21 members and meetings are held monthly on specific dates. In the HMG meetings, Female Community Health Volunteers (FCHVs), Nepal’s most local health system representative, share information and facilitate discussion on a wide range of health topics, including nutrition and maternal and child health. To date, there are more than 52,000 FCHVs in Nepal, each leading one HMG [4–7].

Although HMGs are an essential platform for providing health and nutrition services in Nepal [4], they remain underutilised, with only 27% of eligible women participating in at least one HMG meeting in the last six months of 2016 [3]. The underlying reasons for this low participation rate are unclear. Previous studies have documented that socioeconomic factor such as education, wealth, relationship, and employment status may enable or constrain women’s participation in the voluntary groups [8, 9]. A recent review in India, a context similar to Nepal, having a regular meeting schedule, intergeneration participation (e.g., participating with mother-in-law) and the discussion topics covered influenced participation [10]. These studies demonstrate that individual, intrapersonal, and intervention-related factors may influence participation. However, there is still a gap in understanding why women participate or not in HMG meetings, particularly in Nepal and other low-income settings.

To help fill this gap, inform health promotion policies in Nepal, and contribute to improvements in women and children’s health and nutrition, we examined the facilitators and barriers of HMG meeting participation.

## Methods

### Settings

This mixed-methods study was conducted in Nepal, an LMIC in Southeast Asia, comprised of 77 districts. The quantitative component entailed a secondary analysis of the Nepal Demographic and Health Survey (NDHS) 2016, a nationally representative survey, while the qualitative component entailed collecting data via interviews and group discussions held in two purposively selected sites—one rural municipality (Kaligandaki) and one urban municipality (Chapakot) in Syangja district. The HMG meetings in these settings had a fixed date and venue (7th and 14th of every Nepali month in Kaligandaki and Chapakot respectively). In both municipalities, the HMG meetings usually lasted for two to three hours and were conducted in tandem with other meetings/activities such as antenatal care (ANC) check-ups, women’s development meetings, financial savings programmes, and blood pressure measurements. While Kaligandaki’s HMG meetings were held in a fixed structure venue, women in Chapakot met in the open-air.

### Participants and Data Collection

For the quantitative study component, we used data from the NDHS 2016, which had a response rate of 98.3% [3]. Details about the sample size calculation and sampling methods are described in the NDHS 2016 report [3]. To answer our research question, we extracted NDHS women’s questionnaire data collected among women aged 15–49 years who were aware of HMG meetings in their communities. These data were collected by trained interviewers using structured questionnaires that included caste, women’s age, women’s education, wealth quintile, number of children under five years, household headship, remoteness, family size, health care decision maker, women’s employment status, and participation in HMG meetings [3].

For the qualitative component, we collected primary data by conducting 35 in-depth interviews (IDIs) with 1000-day women, FCHVs and health workers and eight focus group discussions (FGDs) with FCHVs, health workers, and male and female decision-makers separately (methods for these IDIs and FGDs are described elsewhere [11]). The IDIs and FGDs guide questions were formulated to align with the research question and developed based on the literature on mother’s group [6, 8] and the local context of HMGs in Nepal. These guides were also pre-tested and revised, as necessary. The major topics explored with the different categories of study participants were perceptions of the HMG, including meeting status, awareness of the meetings, barriers and enablers for participation, women’s interest in and perceived value of HMGs, and the suggestions for strengthening HMG participation.

### Data management and analysis

In the quantitative analysis, participation in HMG meetings in the last six months was dichotomised as “Yes” if the mother attended at least one or more meetings in the previous six months, and “No” otherwise. Associations between different socioeconomic variables and participation in the HMG meetings in the last six months were assessed using a multivariable logistic regression accounting for sampling weights and sampling design (i.e., stratification and clustering). Standard errors were computed using the linearized variance estimator based on a first-order Taylor series linear approximation [12]. The regression model included women’s age (15–25,26–35,36–45,46–49 age groups), women’s education (no education, primary, secondary and higher schooling), caste (Brahmin/Chhetri, Janajati, Dalit and others), household headship (women and men), wealth quintile (as per the original survey, poorest, poorer, middle, richer and richest), remoteness (rural and urban), number of children under five years of age (none, one or two children and three or more children), women’s employment status (yes and no), family size (less than five and five and above), and health care decision maker (wife alone, husband and wife joint, and husband alone and other family members). These variables were selected considering the existing literature and the local context of Nepal [6, 8]. Since we purposefully limited the data set to women who were aware of HMGs meeting in their ward, we accounted for this subpopulation selection in the analysis. Quantitative analyses were conducted using Stata (version 15) [13] and results were presented as adjusted odds ratios (aORs) with 95% confidence interval (95% CI). Differences with p-values < 0.05 considered significant.

For the qualitative interviews, each IDI and FGD were audio-recorded, transcribed, and translated into English by two independent translators, with quality assurance of randomly selected transcripts conducted by the lead researchers. Analysis was conducted using NVivo 12 (QSR International). Using deductive coding, researchers identified facilitators and barriers to HMG meeting participation from the individual to structural levels. The identified factors were then aligned to the socio-ecological model (SEM) for health promotion framework [14] and similarities and differences were assessed according to study participant groups and data collection sites. After completing the analysis, researchers returned to the study sites to present and validate these findings.

**Fig. 1:**
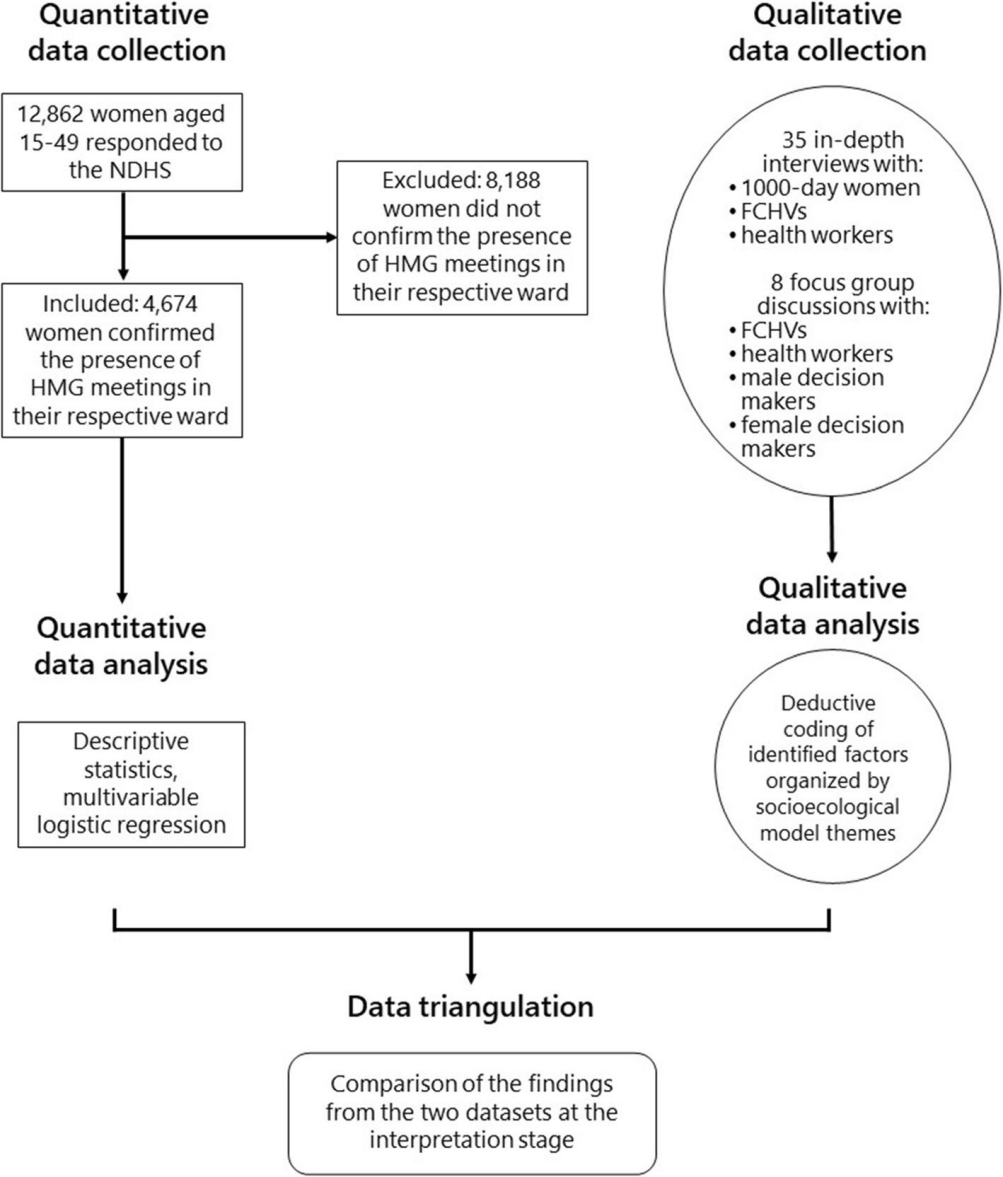
Data triangulation process

To triangulate the data between the two methods, we followed a convergent mixed-method design where we first separately analysed the quantitative and qualitative data sets and then integrated the findings from both datasets when interpretating of the results (Fig. 1). In the integration stage, we compared the qualitative findings with the NDHS survey, and identified areas of convergence (similarity) and divergence (difference) between the two datasets [15].

### Ethical review

 The study was approved by the Nepal Health Research Council, ICF Institutional Review Board and FHI 360’s Protection of Human Subject Committee (PHSC). Informed consent was obtained from all study participants for both interviews and recordings.

## Results

### Study Population and Characteristics

Of the 12,862 women aged 15–49 surveyed in the NDHS, 4,674 confirmed the presence of HMG meetings in their respective ward. Many of these women were Brahmins/Chhetri (relatively advantaged caste, 40.1%) while nearly 23% belonged to the poorest wealth group. The majority were less than 35 years of age (Table 1).

For the qualitative component, a total of 70 individuals participated in 35 IDIs and eight FGDs. IDIs were conducted with twenty 1000-day women, six health facility staffs and nine FCHVs. Two of the eight FGDs were held with health facility staff, two with FCHVs, two with male decision makers and two with female decision makers (Table 2) [11]. Most of the 1000-day women were in their mid-twenties and were Brahmins (70%). Approximately one third of women (35%) completed 10 years of schooling. The mean age of health facility staff was 28 years with most being Janajati (less advantaged caste, 63%). The average age for FCHVs was 51 years. Most FCHVs were Brahmins (65%) and over half (53%) did not complete secondary school (less than eight years of schooling). All the female decision makers were mothers-in-law with an average age of 50 years and the majority of the male decision makers were husbands of the 1000-day women with a mean age of 29 years.

**Table 1:** Demographics of the study population in the NDHS

	n^a^ (%)^b^
Variable	*N* = 4,674
**Caste**	
Brahmin/Chhetri	2,056 (40.1)
Janajati	1,667 (36.9)
Dalit	580 (11.9)
Other	371 (11.2)
**Women’s age in completed years**	
15–25	1820 (37.7)
26–35	1440 (31.4)
36–45	1082 (23.7)
46 and above	332 (7.2)
**Women’s education**	
No Education	1,658 (34.9)
Primary	689 (15.7)
Secondary	1725 (36.1)
Higher	602 (13.2. )
**Wealth quintile**	
Poorest	1,282 (22.9)
Poorer	1,108 (22.3)
Middle	963 (21.2)
Richer	784 (19.2)
Richest	537 (14.4)
**Number of children under five years**	
None	2,476 (53.6)
1 or 2 children	2,036 (43.7)
3 or more	162 (3.4)
**Household headship**	
Female	1555 (33.1)
Male	3119 (66.9)
**Remoteness**	
Urban	2858 (59.8)
Rural	1816 (40.2)
**Family size**	
Less than five	1924 (42.3)
Five or above	2750 (57.7)
**Health care decision maker**	
Women alone	920 (19.7)
Husband and women joint	1155 (27.3)
Husband alone or other family members	2599 (53.0)
**Currently employed**	
No	1472 (32.5)
Yes	3202 (67.5)
^a^Unweighted frequencies, ^b^weighted percentage	

**Table 2:** Description of the qualitative sample

Approach	N	Description
**In-depth interviews**	35	
Health facility staff	6	Three per site
FCHVs	9	Four in Kaligandaki and five in Chapakot
1000-days women	20	10 per site
**Focus group discussion**	8	
Health facility staff	2	One per site with four participants per FGD
FCHVs	2	One per site with four participants per FGD
Male decision makers	2	One per site with four to five participants per FGD
Female decision makers	2	One per site with five participants per FGD

### Quantitative findings

Table 3 shows the associations between socioeconomic factors and participation in HMG meetings. Women with children, above 26 years of age, with formal schooling, employed, poorer based on wealth quintile, living in male headed households, and from rural areas were found to be significantly associated with an increased odds of participation in HMG meetings. Women from the Janajati and other castes were significantly less likely to participate in HMG meetings compared to women from the Brahmin/Chhetri caste. Women were also less likely to participate when health decisions were made by the husband or other family members. Family size was not significantly associated with participation.

**Table 3:** Associations between socioeconomic factors and participation in the HMG meetings

Socioeconomic factors	Participated	Not Participated	aOR^a, b^ (95% CI)
	**N (%)** ^a^	**N (%)** ^a^	
**Number of children under five years of age**			
None	485 (21.9)	1735 (78.1)	1
1 or 2 children	523 (29.3)	1262 (70.7)	**1.67 (1.41–1.98)**
3 or more	53 (37.6)	88 (62.4)	**2.81 (1.88–4.19)**
**Women’s age in completed years**			
15–25	296 (18.9)	1360 (81.8)	1
26–35	393 (30.3)	906 (69.7)	**1.96 (1.57–2.44)**
36–45	297 (30.2)	686 (69.8)	**2.63 (2.00-3.46)**
46 and above	75 (25.2)	223 (74.8)	**1.95 (1.35–2.82)**
**Women’s education**			
No Education	392 (27.1)	1057(72.9)	1
Primary	184 (28.2)	468 (78.1)	**1.33 (1.03–1.71)**
Secondary	365 (24.4)	1133 (75.6)	**1.63 (1.26–2.11)**
Higher	119 (21.8)	428 (78.2)	**1.50 (1.11–2.03)**
**Household headship**			
Female	324(23.6)	1049(76.4)	1
Male	736(26.6)	2036(73.4)	**1.31 (1.09–1.57)**
**Caste**			
Brahmin/Chhetri	492 (29.6)	1169 (70.4)	1
Janajati	344 (22.5)	1186 (77.5)	**0.69 (0.56–0.85)**
Dalit	133 (27.0)	358 (63.0)	0.85 (0.63–1.14)
Other	93 (20.0)	370 (80.0)	**0.65 (0.46–0.91)**
**Remoteness**			
Urban	551 (22.2)	1928 (77.8)	1
Rural	510 (30.6)	1157 (69.4)	**1.31 (1.05–1.65)**
**Wealth quintile**			
Richest	98 (16.5)	500 (83.5)	1
Richer	172 (21.5)	626 (74.9)	**1.53 (1.05–2.23)**
Middle	222 (25.2)	656(74.8)	**1.98 (1.41–2.78)**
Poorer	257 (27.8)	667(72.2)	**2.27 (1.58–3.25)**
Poorest	313 (32.9)	636(67.1)	**2.59 (1.80–3.72)**
**Family size**			
Less than five	435 (24.8)	1319 (75.2)	1
Five and above	626 (26.2)	1765 (73.8)	0.96 (0.80–1.15)
**Health care decision maker**			
Wife alone	245 (30.0)	571 (70)	1
Husband and wife joint	343 (30.3)	790 (69.7)	1.05 (0.83–1.33)
Husband alone and other family members	473 (21.5)	1723 (78.5)	**0.69 (0.55–0.86)**
**Currently employed**			
No	266 (19.7)	1083 (80.3)	1
Yes	795 (28.4)	2002 (71.6)	**1.28 (1.05–1.56)**
^a^Weighted percentages and aORs ^b^Multivariable model adjusted for caste, women’s age, women’s education level, wealth quintile, number of children under five years of age, household headship, remoteness, health care decision maker, women currently employed, and family size Bolding indicates P value < 0.05. aOR = adjusted odds ratio, 95% CI = 95% confidence interval			

### Integrating qualitative results with quantitative findings

The qualitative results in this section are integrated with the quantitative findings and presented according to three levels of the SEM (individual, interpersonal, and community), from the most to least proximate.

### Individual level: Hopes and perceptions regarding the HMG meetings

At the individual level, HMG meeting participation was affected by women having young children in the household, women’s interest in acquiring new knowledge, their age and educational status and women’s advanced awareness of the meeting schedule and venue.

### *Young children in the household*

From the perspective of the FCHVs and male decision makers, women’s interest in participating in HMGs was driven by a desire to gain knowledge about their child’s health. Mothers of young children were said to be eager to obtain information related to nutrition, immunisation, sanitation and hoped to gain knowledge and skills both for themselves and for their children. Similar findings were observed with the quantitative survey data, which showed that women who had three or more children under 5 years of age were 2.81 times more likely to participate (aOR = 2.81; 95% CI: 1.88–4.19) when compared to women who did not have children.

*Since there is more focus on the topic of how to prevent children from malnutrition and what should be done in order to keep them healthy, they come and attend the meeting.* (FCHV, Chapakot, FGDs)

### *Age*

The responses from the interviews were divergent with the quantitative survey for women’s age. According to some FCHVs, women’s age affected participation, with older women perceived to be less willing to join HMG meetings compared to younger women because older women consider the health-related information provided to be more useful to younger mothers.

*In my opinion, the old mothers may feel that health related information is not for them but for young people, so they may not have come. The younger women come.* (FCHVs, Kaligandaki, IDI)

### *Education levels*

While quantitative data showed that HMG participation increased with education levels, the qualitative results were mixed. Some FCHVs expressed concerns that that the difference in literacy between themselves and more educated women hindered HMG meeting participation. This was said to be particularly true for better educated women who were perceived as knowing more than FCHVs and thus would not benefit from the sessions.

*It is difficult to bring educated people near. They are more educated than us and have studied up to class 11, 12. They think that we do not know as much them. The educated people say that they know more than us.* (FCHVs, Kaligandaki, IDI)

However, according to some health workers and other FCHVs, having an education encouraged women to participate in the HMG meetings because women wanted to learn.

*Most of them are educated and they have learned some things in the school. They are more qualified than us, but still they come.* (FCHVs, Chapakot, IDI)

*The educated are interested in new things and want to be involved in HMGs.* (HW, Chapakot, FGD)

### *Women’s interests in acquiring new knowledge*

This factor was only captured in the qualitative findings. Women who joined the HMG meetings expressed an interest in the health information provided during the meetings and reported that they gained awareness on hygiene, cleanliness, nutritious food preparation and child feeding, maternal and child health, iron and vitamin intake and other topics.

*We get to ask what we have in our mind and get to know how to feed our baby to make him healthy. In previous month, we got to know about Baal Vita, lito* [nutritious food] *and I knew that they would teach ways to prepare it, so I went.* (1000-day women, Kaligandaki, IDI)

### *Advanced awareness of the meeting schedule and venue*

This factor was not available in the quantitative data; however, in the qualitative data women and their family members perceived the irregular meeting schedule, and lack of timely reminders of the HMG meeting as a barrier to participation. Some 1000-day women from both study sites reported that the FCHV did not inform them about the meeting while male decision makers from Kaligandaki noted that 1000-day women were not well informed about the meeting dates, times, venues or contents, which discouraged them from attending.

*When they* [1000-day women] *know, they go. Sometimes they* [FCHVs] *call by phone when she* [1000-day women] *has gone to cut grasses. At 9 am they inform that there will be meeting at 10 am. But at that time, she may be in the hay field and unable to walk that long distance* [to reach the meeting on time]. (Male decision makers, Kaligandaki, FGD)

### Interpersonal level: Family hierarchy and socio-cultural norms

At the interpersonal level, family support was identified as an enabler of HMG participation while work obligations and caste discrimination hindered engagement.

### *Family support*

According to some 1000-day women and FCHVs, family/husband/mother-in-law support is a prerequisite for women to participate in the HMG meetings. Many of the 1000-day women from both municipalities reported having this support.

*I want to go and my* [family] *allows me to go to such health-related programmes so that I would gain knowledge related to taking medicines and vitamins. They* [family] *also allow me to go when information regarding proper care of babies is given. They do not allow me to go other times.* (1000-day women, Chapakot, IDI)

Quantitative data provided evidence that family support is important. In the survey, women living in a male-headed households had a 1.31-fold increase in participation (aOR = 1.31; 95% CI:1.09–1.57) compared to women living in female-headed households.

### *Caste*

The qualitative interview responses were convergent with the quantitative results for caste. FCHVs indicated that the Dalit and Janajati communities were perceived as illiterate and uninterested in attending the HMG meetings, hindering their participation. Although HMG meeting participation varied across different castes, both FCHVs and 1000-day women indicated that women from any caste were welcome to participate in the HMG meetings.

*They* [Dalit and Janajati] *are usually illiterate and unaware…. So, they don’t come much…. it is quite difficult to make them understand.* (FCHVs, Kaligandaki, IDI)

### *Work obligations during the agricultural season*

This data point was not captured in the survey results, but from qualitative data, according to 1000-day women, FCHVs and health facility staff, work obligations served as a major barrier to HMG participation. In particular, agricultural seasons (July-September and December/January) were identified as a time when women were too busy with additional household work, such as preparing snacks for field workers, to be able to participate. In Chapakot, FCHVs reported rescheduling meetings because women are busy during the planting season, while in Kaligandaki, FCHVs acknowledged that although participation declines during the planting seasons, they did not reschedule meetings.

*If the agriculture work is on the 7th* [HMG meeting day] *it is not possible to attend the meeting.* (1000-day women, Kaligandaki, IDI)

### Community level: services and infrastructure

At the community level, only qualitative data were available for analysis. These data showed that having additional services offered during HMG meetings and engaging with health facility or non-governmental organisation staff facilitated women’s participation while the lack of meeting structures served as a barrier.

### *Additional services*

1000-day women and FCHVs reported that when HMG meetings were combined with other activities or services—provision of blood pressure measurement, distribution of lito (nutritious food), and savings/financial programming—women were more likely to attend because they were able to complete both/all activities at once. This was especially true for Kaligandaki, a, rural municipality where women live far from the HMG meeting location.

*We have kept the ANC checkup* [additional services] *on the same day* [of the HMG meeting] *as it will be easy for the ones who are staying far away* (1000 day women, Kaligandaki, IDI)

*We motivated them … by letting them know that we have services like measurement of blood pressure. They would* [typically] *have to go far to have their blood pressure measured, which we provide here. So we told them to come to measure blood pressure and also listen to the discussion since they would be able to learn a lot of things.* (FCHV, Kaligandaki, IDI)

### *Engagements with external facility staffs*

In both settings, the engagement of health facility staff and frontline workers from non-governmental organisations was identified as a facilitator for women’s participation in HMG meetings. 1000-day women, health facility staff and FCHVs highlighted the benefit of having frontline workers conduct regular meetings and facilitate additional activities, for example food demonstrations. In addition, in Chapakot, FCHVs indicated that facilitation support from health facility staff increased women’s participation and interest in the meeting.

*There is more participation if sirs and sisters are there* [referring to heath facility staff in the HMG meeting]… *People pay more attention when someone from the health facility facilitates the meeting and provide new information every month…We conduct the meeting together and people believe more when we speak with the support of health facility staff.* (FCHV, Chapakot, IDI)

### *The meeting infrastructure*

An important barrier identified by FCHVs, 1000-day women and health workers in Chapakot was the lack of a structure for meetings, especially during the rainy seasons. In contrast, Kaligandaki FCHV reported that they face no challenges in conducting the HMGs meetings even during the rainy season because meetings are held indoors.

*If it rains, there is no place to meet because here is no building for the meeting, if it doesn’t rain, we meet. If it is raining, we tell people we will be there by 11, but if it keeps raining, we try to get there at 1–2 and still get a few things done in an hour or two. But if it rains the entire day, we cancel the meeting.* (Health facility staff, Chapakot, FGD)

*Regarding venue, we have our own building constructed so, there are no problems regarding that. There is no problem even when there is rainfall.* (FCHV, Kaligandaki, IDI)

## Discussion

Our mixed-methods study identified the facilitators and barriers of HMG meeting participation according to three levels of the socio-ecological model: individual, interpersonal and community.

At the ***individual level***, our findings revealed that women’s interest in gaining knowledge was a key facilitator of meeting attendance because women were interested in gaining knowledge about their own and their children’s health from the HMG meetings. Our qualitative and quantitative results also suggest that the meetings are more valuable to women with young children. This may be because the HMG meetings specially target women in the 1000-day period.

Interestingly, the quantitative and qualitative results related to the influence of education were not aligned. According to the quantitative data, more educated women were more likely to participate in HMGs while the qualitative results suggest that educated women perceive FCHV’s limited health literacy as a barrier to meeting attendance. Similar results were found in a previous study where the education and age gap between educated young women and FCHVs negatively influenced the uptake of services provided by FCHVs [16]. Another study suggested FCHVs’ lower education level may affect their ability to communicate health messages [17] potentially reducing women’s trust in the health information provided during the HMG meetings. Improvement in the quality of training, ongoing refresher courses and mobile health technology can improve FCHVs knowledge, communication, and quality of their interaction with participating women [17, 18]. Indirectly, these interventions may strengthen women’s trust in the health information provided in the HMG meeting and increase their participation. In addition to improving FCHV’s ability to conduct effective HMG meetings, it would be valuable to explore further why educated women attend the meetings and identify any additional facilitators among this population.

Although HMG meetings are considered an essential platform for maternal and child health in Nepal, our study identified irregular meeting schedules and the lack of untimely meeting reminders as a major barrier to participation. According to a systematic review of women’s participation in women’s groups in India, women were more likely to participate if meetings were held regularly over an extended period, while meetings conducted irregularly discouraged women’s participation [10]. To mitigate this challenge, text messages delivered via mobile phones could provide women with accurate meeting information (e.g., date, time, purpose, and planned discussion topics). This appears to be a relevant solution because according to a qualitative study in Nepal, many 1000-day women have a mobile phone, can read text messages, and expressed interest in receiving text message reminders on HMG meeting dates, time, and discussion topics [11]. However, further validation of this approach as well as identifying solutions for women without access to mobile phones and texting abilities in the broader context of Nepal are needed.

At the ***interpersonal*** level, both the quantitative and qualitative data identified family support, particularly from mothers-in-law and husbands, as an important enabler of HMG meeting participation. This finding is consistent with previous studies investigating barriers to service uptake, including services provided by FCHVs, which found that younger Nepali women have limited decision-making autonomy [17, 19], while mothers-in-law have a strong influence on daughters-in-law health service uptake [20]. Based on this finding, HMG meeting promotion efforts should advocate mothers-in-law and husbands to support women’s participation. However, this is likely insufficient for securing women’s HMG participation because, according to our results, women’s need to do additional household work during the agricultural season was a barrier to participation. The requirement for additional work is likely explained by Nepal’s gender and social norms that require women to take on extra chores [21]. Interestingly, other studies have found women’s limited power in the agricultural sector and highlighted the importance of empowering them and securing their leisure time, particularly for child health outcomes [22]. This suggests that familial support needs to extend giving beyond permission to relieving women of additional tasks during the agricultural season so they can participate in HMG meetings throughout the year.

At the ***community level***, the qualitative analysis showed that additional services may help to improve HMG meeting participation, particularly for women in Kaligandaki (a rural municipality) who are living far from meeting venue. And though previous studies have shown that residents of rural areas in Nepal may have less access to health and education services compared to those in urban areas [23] due to distance and poor road conditions [24], this may not be the case for HMG meetings. According to the survey data, rural women were more likely to participate than women living in urban areas. This may be because rural women more highly value HMG meetings since health resources are scarce in rural Nepal[23]. It may also be because additional services such as blood pressure and food distribution, offered alongside the HMG meetings in rural areas, may encourage the participation of women who live far from meeting venues since they complete a range of activities at once, reducing opportunity costs. However, it is important to note that not all HMG meetings in rural area offer additional services, thus there may be other reasons rural women participate more than the urban women.

 Another enabling factor of meeting participation according to our study was the engagement of health facility staff and other frontline workers as meeting facilitators. This may be because women trust health facility staff. It may also be because these staff use different facilitation techniques than other cadres or cover a wider range of discussion topics while facilitating HMG meetings. According to a 2018 review, supportive supervision and continued support from community health volunteers’ managers was critical to strengthening volunteer performance and the successful delivery of health services in LMICs [25]. Combined, these findings suggest that health facility staff and other frontline workers can play an important role in improving HMG participation.

As with many studies, this research has several limitations. Because we conducted a cross-sectional analysis of the quantitative data, we were unable to determine causality. In addition, this analysis was restricted to women who confirmed awareness of HMGs, potentially introducing a selection bias. Future quantitative studies designed to include all reproductive age women irrespective of awareness of HMG meetings could mitigate this shortcoming. Qualitative data were limited to two sites, which means the results were not representative of the Nepali context overall and may not reflect the national sample of the quantitative data. In addition, not all variables, such as travelling distance to meeting venues, were available in the quantitative dataset, so we were unable to assess these associations. Despite these limitations, by using a mixed-methods design and triangulating the results, our study offers a more comprehensive and deeper understanding of the facilitators and barriers to HMG meeting participation by examining both the strength of the associations and potential explanations for the associations observed. Furthermore, the NDHS 2016 was a national representative survey with a relatively high response rate, and our sample for the qualitative analysis was relatively large, including 70 study participants in 35 IDIs and eight FGDs with 35 participants.

## Conclusions

According to our study, women’s participation in HMG meetings in Nepal is facilitated and hindered by factors at the individual, interpersonal and community levels of the SEM. To improve women’s participation in HMGs in Nepal, it is necessary to enhance FCHV literacy, provide advance notice of the meeting schedule, and secure stable and upgraded meeting venues. Familial support to help with women’s workload to allow their participation, particularly during agricultural seasons, is also needed. Further, combining meetings with key services will likely increase participation, especially for rural women. Our findings are essential for effective implementation of the HMG meetings and similar women’s group-based platforms, and ultimately improving maternal and child health in Nepal.

## Data Availability

The quantitative datasets used for the current study are available from the DHS program (http://www.dhsprogram.com/data/available-datasets.cfm) on request. The qualitative dataset is not publicly available but are available from the corresponding author on reasonable request.

## Abbreviations

ANC: Antenatal care
LMICs: Low and middle-income countries
HMG: Health mothers’ group
FCHV: Female community health volunteers
NDHS: Nepal demographic health survey
IDI: In-depth interview
FGD: Focus group discussion
SEM: Socio-ecological model
PHSC: Protection of Human Subject Committee

## Declarations

The qualitative study was approved by the Nepal Health Research Council on July 2, 2018, and Family Health International (FHI) 360’s Protection of Human Subject Committee (PHSC) on November 21, 2018. Informed consent was obtained from all study participants for both interviews and recordings. Individual’s autonomy and confidentiality was ensured throughout the research process.

All the methods were carried out in accordance with the Nepal Health Research Council and Family Health International (FHI) 360’s Protection of Human Subject Committee (PHSC) guideline.
